# Nitrogen implantation with a scanning electron microscope

**DOI:** 10.1038/s41598-017-18373-z

**Published:** 2018-01-08

**Authors:** S. Becker, N. Raatz, St. Jankuhn, R. John, J. Meijer

**Affiliations:** Division of Nuclear Solid State Physics, Felix Bloch Institute for Solid State Physics, Faculty of Physics and Earth Sciences, Universität Leipzig, Linnéstraße 5, D-04103 Leipzig, Germany

## Abstract

Established techniques for ion implantation rely on technically advanced and costly machines like particle accelerators that only few research groups possess. We report here about a new and surprisingly simple ion implantation method that is based upon a widespread laboratory instrument: The scanning electron microscope. We show that it can be utilized to ionize atoms and molecules from the restgas by collisions with electrons of the beam and subsequently accelerate and implant them into an insulating sample by the effect of a potential building up at the sample surface. Our method is demonstrated by the implantation of nitrogen ions into diamond and their subsequent conversion to nitrogen vacancy centres which can be easily measured by fluorescence confocal microscopy. To provide evidence that the observed centres are truly generated in the way we describe, we supplied a 98% isotopically enriched ^15^N gas to the chamber, whose natural abundance is very low. By employing the method of optically detected magnetic resonance, we were thus able to verify that the investigated centres are actually created from the ^15^N isotopes. We also show that this method is compatible with lithography techniques using e-beam resist, as demonstrated by the implantation of lines using PMMA.

## Introduction

The development of the electron microscope in the 1930s was a milestone in many fields of natural sciences^1^. Its high resolution enabled scientists to investigate very small objects like cells or large molecules. The wide area of application and the gradually dropping costs of electron microscopes led to the fact that today most natural science institutes have such a device. In this research paper we report about another application of the electron microscope apart from its imaging technique, that was to our best knowledge not published up to now. We demonstrate here that the implantation of ions is feasible by the simple means of a scanning electron microscope (SEM) only. Usually an ion accelerator is needed for a precise implantation of atoms, which is not available in many research facilities because of the technical complexity and correspondingly high costs.

The proof of principle of our concept is done with nitrogen atoms/molecules and their conversion and detection as nitrogen vacancy (NV) centres^2^. If not explicitly stated otherwise by writing NV centre we refer to the negatively charged type (NV^−^). The NV centre consists of a substitutional nitrogen atom next to a vacancy in the diamond lattice. The centre has a zero phonon optical transition at a wavelength of 638 nm^3^ between its triplet ground state ^3^A_2_ and its first excited state ^3^E, which is also a spin triplet. In addition, there is an other decay path comprising a metastable singlet state. The ground state triplet has a splitting at zero magnetic field of 2.87 GHz^4^ between the *m*_S_ = 0 and *m*_S_ = ±1 electron spin sublevels. This energy level constitution opens the possibility for the initialization, manipulation and read out of the centres quantum state and therefore its employment as a qubit^5–7^. The method described here is highly relevant for the research work on NV centres because nitrogen implantation into ultrapure diamond^8^ is a often used technique for the generation of NV centres.

## Results

### Identification of NV centres

**Figure 1:**
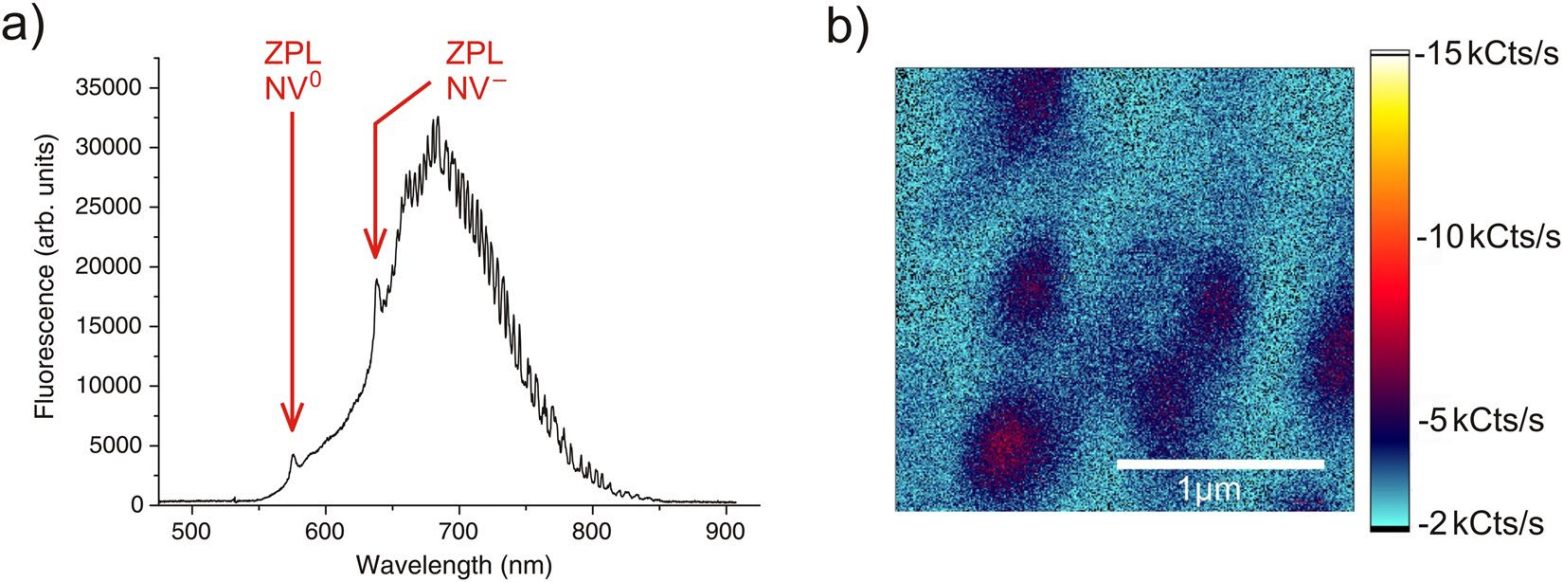
Signal of the created NV centres. (**a**) Fluorescence spectrum recorded in the centre of the area that was irradiated with the electron beam of the SEM (front side of the sample). The ZPLs of NV^−^ (638 nm) as well as of NV^0^ (575 nm) are clearly observable. (**b**) Fluorescence image of individual centres near the irradiated area.

To demonstrate our method a rectangular area on the front side of an electronic grade diamond (${{\rm{N}}}_{{\rm{s}}}^{0}\le 5$ ppb) was irradiated with a low energy electron beam in a SEM. The SEM was equipped with an additional gas inlet, so that isotopically enriched nitrogen (98% ^15^N) can be directed into the chamber. After the diamond was annealed in vacuum to form NV centres, fluorescence images and spectra of the sample were recorded. Fig. 1a) shows a fluorescence spectrum of the central region of the electron beam irradiated area of the sample. The zero phonon line (ZPL) of NV^−^ is clearly visible at 638 nm. Additionally, the ZPL of the neutrally charged NV centre (NV^0^) can be seen at 575 nm. Both are accompanied by their characteristic phonon side bands. These spectra could be recorded everywhere in the irradiated area, proving that the generated defects are really NV centres. No NV centres could be found in the other sectors of the sample. But a lower fluorescence signal could be measured around the irradiated area, quickly dropping down to the point where individual centres were resolvable. A possible explanation for this effect is discussed below. Figure 1b) shows a fluorescence image of these single centres near the irradiated area. Conducted photon antibunching measurements^9^ and taken spectra verified that the fluorescing spots were indeed single NV centres.

### Identification of nitrogen-15

**Figure 2:**
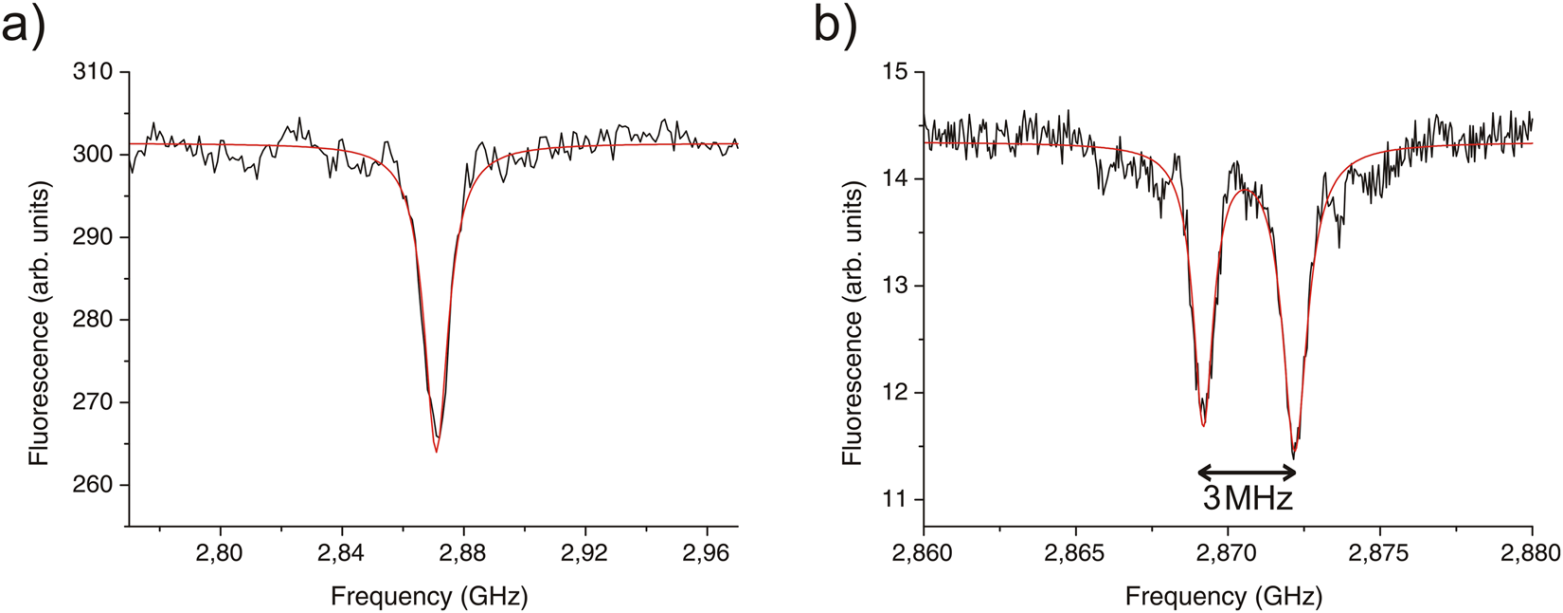
ODMR spectra of an individual NV centre at zero magnetic field. (**a**) The centre was simultaneously excited with microwaves and laser light. A single dip is visible at 2.87 GHz, characteristic for a NV centre. (**b**) The centre was excited by a *π*-pulse followed by optical illumination. A dual separation of the 2.87 GHz resonance is observable. The fit yields a magnitude of 3 MHz corresponding to the hyperfine splitting expected for a ^15^NV centre.

The generated NV centres were also investigated with optically detected magnetic resonance (ODMR) measurements to identify whether their constituting nitrogen atoms are of the isotope type ^14^N or ^15^N. Their distinction is possible because of the different nuclear spin of ^14^N (*I* = 1) and ^15^N (*I* = 1/2) that leads to a differing hyperfine coupling to the electron spin (*S* = 1) of the NV centre. To make a statistically sound statement, the ODMR spectra of 49 centres were recorded in a batch measurement. Figure 2 depicts two ODMR spectra for one of these centres, where the detected fluorescence is plotted over the irradiated microwave frequency. Figure 2a shows the continuous wave (cw) spectrum of this centre over a range from 2.77 GHz to 2.97 GHz. The cw spectrum exhibits the expected behavior for a NV centre at zero magnetic field with the single dip in fluorescence at 2.87 GHz. This resonance corresponds to the microwave excited transition between the *m*_S_ = 0 and *m*_S_ = ±1 electron spin sublevels. In Fig. 2b a pulsed spectrum of the same NV centre is depicted. Here the 2.87 GHz resonance is further investigated in a range of 20 MHz. The spectrum shows the typical dual separation of the dip that is expected for the hyperfine interaction between a nuclear spin of *I* = 1/2 and the electron spin of *S* = 1 of the NV centre at zero magnetic field. This hyperfine interaction leads to a twofold splitting of the electron spin sublevels and hence the dual separation of the dip in the fluorescence. The magnitude of this hyperfine splitting is determined by a fit of a Lorentzian function to the data that yields a separation of 3 MHz.

**Figure 3:**
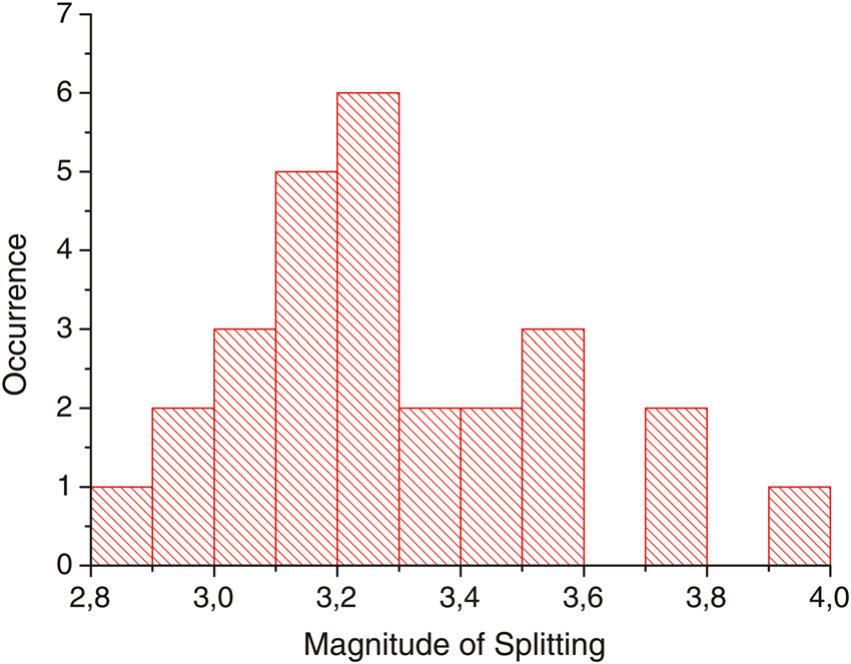
Histogram depicting the magnitude of the twofold splittings of the identified ^15^NV centres which ranges from 2.85 MHz to 3.95 MHz.

This value is in good agreement with data from the literature^10^ for the hyperfine splitting in the ground state of a NV centre that comprises a ^15^N atom. In the case of the constituting nitrogen atom of the NV centre is a ^14^N isotope, a threefold splitting of the electron spin sublevels and hence in the dip in the fluorescence would be expected. The magnitude of this splitting is stated in the literature as −2.16 MHz^11^. Additional splittings due to the hyperfine interaction of the NV electron spin with other nuclear spins are possible and were indeed observed in some of the recorded spectra. Because only a nuclear spin of *I* ≠ 0 gives rise to hyperfine interactions, the most common nuclei for this interaction are ^13^C isotopes. For a profound treatment of this matter we refer to the corresponding literature^12^. Figure 3 shows a histogram of all measured centres that could be identified as ^15^NV centre grouped by the magnitude of their splitting. All these centres have a twofold splitting of the 2.87 GHz resonance that ranges from 2.85 MHz to 3.95 MHz.

### Investigation of generated structures

**Figure 4:**
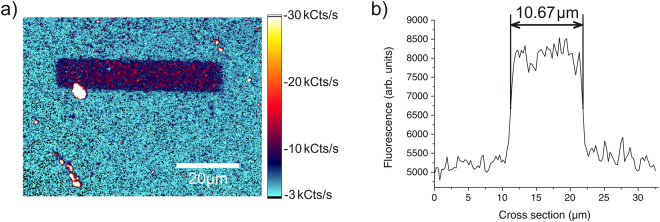
Line composed of NV centres. (**a**) Fluorescence image of a spot where one of the openings of the PMMA mask has been (back side of the sample). At the former location of the opening numerous NV centres have been created, whose fluorescence signal is clearly visible against the background. (**b**) Averaged fluorescence along the cross section of the line shown in (**a**) and the surrounding background.

Following the analysis of the electron irradiation process on the front side of the sample, an electrically insulating PMMA mask comprising a set of lines and circles was created on the surface of the back side by electron beam lithography. The sector of the diamond containing the openings was subsequently irradiated with the electron beam of a SEM with no gas inlet. After the annealing process, confocal investigations of this sector showed an increased signal at the spots were the openings of the mask have been. Again recorded spectra confirmed that the detected fluorescence originated from NV centres. Figure 4a shows a fluorescence image of one of the created lines, where the line itself is sharply delimited from the background. The white spot at the left side of the line’s lower edge is a contamination. In Fig. 4b a graph depicts the averaged fluorescence along the cross section of this line and the surrounding background (without the contamination). From this the width of the line is determined as 10.67 *μ*m, which is fairly close to the intended value of 10 *μ*m. While the lines exhibited a moderate density of NV centres, the circles only featured a very low density of NV centres. In fact only the signal of one set of circles that consisted of a 10 *μ*m, a 5 *μ*m and a 3 *μ*m circle could be located with the confocal microscope. The signal was so weak that it was hardly distinguishable from the background.

## Discussion

**Figure 5:**
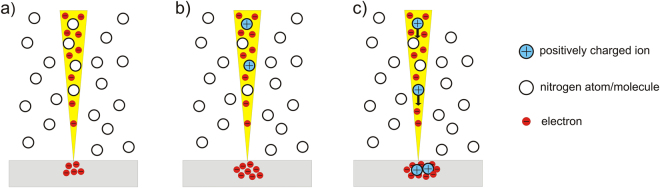
Illustration of ion implantation with a SEM. In (**a**) the electron beam of the SEM is focused on the sample, which leads to an accumulation of electrons due to the insulating sample material. In (**b**) positively charged ions are created by impact ionization of nitrogen molecules and the electrons of the beam. In (**c**) these ions are attracted and subsequently implanted by the negative potential that was built up by the SEM electrons in the sample.

The mechanism for ion implantation via a scanning electron microscope is depicted in Fig. 5 and can be described as follows: The electron beam of the SEM is focused onto the sample. Due to the electrically insulating character of the diamond, the electrons can not run off and start to accumulate in it. As a result, a negative electrical potential builds up that causes an electric field. Because this field also repels the electrons of the beam, the potential however only builds up to the point when it is equal to the acceleration voltage of the electron gun of the SEM. Furthermore, the ^15^N molecules which are led into the chamber and therefore to the sample by the gas inlet (or in the case of the back side irradiation simply the ^14^N residual gas molecules) interact with the electrons of the SEM beam by impact ionization yielding nitrogen ions. The positively charged ions then get attracted by the negative potential in the sample. On the acceleration track they obtain a kinetic energy corresponding to the accelerating voltage. With the obtained energy, the ions are now able to penetrate into the sample and interact with the atoms of the sample material e.g. generating vacancies like in conventional ion implantation. While some of the molecular ions are already dissociated by the impact ionization process, the rest of them breaks apart during the collision with the sample surface. In a subsequent annealing process the vacancies become mobile and thereby enabling NV centre formation^13^.

Even so the method was demonstrated by the implantation of nitrogen ions into diamond, it is expected that because of the underlying physical processes (impact ionization, coulomb interaction) it will work for any other ion species in the gaseous state and also for other insulating substrates. Since the kinetic energy of the ions is limited by the accelerating voltage of the electron gun of the SEM, energies from less than 1 keV up to 30 keV should be feasible. In 27 of our 49 recorded ODMR spectra the observed hyperfine splitting could be clearly assigned to NV centres which are constituted of a ^15^N isotope. No spectra could be assigned to NV centres comprising a ^14^N isotope. That corresponds to a ratio of at least 55.1% of ^15^N containing centres. Considering the natural abundance of the ^15^N isotope of 0.364%^14^, nitrogen atoms that got into the sample during the manufacturing process or their incorporation during other steps of the experiment by accident can be ruled out as the source of the ^15^NV centres. The conducted experiments prove that the generated NV centres are truly formed from the implanted ^15^N ions and therefore that our concept is correct.

We attribute the deviation of the magnitude of the splitting of some centres from the literature value to the particular surface proximity of the NV centres, that arises from the low implantation energy of the nitrogen ions (≤700 eV). Because near the surface the centres are exposed to strain which influences the magnitude of the splitting^15^. Furthermore, a spectral broadening was observed for some centres that is probably induced by paramagnetic impurities which are located at the surface as well^16^. The remaining 22 centres that could not be assigned probably either had such a disturbing environment that their spectra were too much deformed to reliably assign them to ^15^NV or ^14^NV centres or the tracker simply failed to keep track of them. Nevertheless, the majority of the centres exhibit fluorescence and ODMR spectra that are comparable to those of NV centres created by classical ion implantation^8,10,17^.

The observation that a lower density of NV centres was also generated around the area which was irradiated with electrons can be explained by the presented implantation mechanism as well. Because the delivered nitrogen molecules distribute equally in the chamber of the SEM and will have different directions of movement, the vector of motion of some of the nitrogen particles will confine a smaller angle with the sample surface then others. If the angle is small enough the deflection of the particle, that is induced by the electron generated potential in the sample, will not suffice to direct it on a trajectory where it hits the sample in the irradiated area. The ion will instead collide with the surface only when it has already passed the irradiated area, so that it is implanted a bit away from it.

## Conclusion

In conclusion, we have introduced a new method for the implantation of ions into electrically insulating material. This method is based on the impact ionization of gaseous particles, which are brought into interaction with an electron beam and subsequently attracted by a potential induced by the same electron beam, which eventually leads to the implantation of the ions into the insulating sample. It stands out from other established implantation techniques because of its technical simplicity and the associated low costs. The proof of principle was done with nitrogen atoms/molecules that could be verified through generated nitrogen vacancy centres. While fluorescence spectra show that the created colour centres are indeed NV centres, the recorded ODMR spectra prove by identification of the ^15^N isotopes in the centres, that the way the N atoms got into the sample material is as we described it. We also showed that the location of the implanted ions can be restricted to small areas by using an electrically insulating PMMA mask.

## Methods

An electronic grade, single crystal diamond, grown by chemical vapor deposition, was used as a sample in this experiment. The manufacturer, Element 6, stated the initial nitrogen content as below 5 ppb. In a first step the diamond was cleaned in an ultrasonic bath of acetone followed by isopropyl alcohol. Then a 661 *μ*m × 516.4 *μ*m large area on the front side of the sample was irradiated with a low energy electron beam (*E*_kin_ = 700 eV) with a beam current of 35 pA for about 100 hous in a SEM (DSM 982 GEMINI Zeiss), which was modified with an additional gas inlet. The gas inflow, which comes from a metallic cylinder filled with isotopically enriched nitrogen (98% ^15^N, euriso-top) is precisely controlled with a needle valve. The gas is then guided via a silicone tube to a copper ring that is placed between the beam exit and the sample. Six small holes were drilled on the lower part of the inner face of the ring to direct the gas onto the sample. The pressure in the chamber was regulated to 6⋅10^−6^ mbar. After the implantation process the sample was annealed in vacuum at 800 °C for 2 hours to trigger the NV centre formation and subsequently cleaned by etching in a mixture of nitric acid, sulfuric acid and perchloric acid at boiling point for 4 hours. Fluorescence images and spectra of single NV centres and NV ensembles were recorded with a home build confocal microscope equipped with single-photon counting modules and a CCD supported grating spectrometer using a 0.95 NA air objective and a laser light wavelength of 532 nm. Additionally, spectra of ODMR measurements of the ground state of single centres were recorded with another setup that uses the same laser light wavelength and a 1.35 NA oil objective. ODMR is based on a decrease in the emitted fluorescence intensity of the centre caused by the depopulation of the *m*_S_ = 0 state that is achieved by scanning microwaves in an appropriate frequency range. The ODMR data were recorded without applying a magnetic field (*B* = 0) and includes continuous wave spectra (simultaneous microwave and optical excitation) as well as pulsed spectra (applying a *π* pulse followed by optical illumination).

After the evaluation of the first electron irradiation process with the optical setup, the sample was once again cleaned in an ultrasonic bath of acetone followed by isopropyl alcohol. Then the back side of the sample was spin coated with a PMMA electron beam resist at 3500 rpm for 30 s and another 30 s at 3500 rpm down to 0 rpm. Following this a set of lines (width of 10 *μ*m) and circles (diameter of 10 *μ*m to 0.5 *μ*m) was drawn by exposing the sample to a beam of focused electrons at the dual beam microscope Nova NanoLab 200 from FEI and subsequent developing. This procedure formed an electrically insulating mask consisting of PMMA on the sample surface. Afterwards, in the sector of the diamond comprising the set of structures, a 400 *μ*m × 300 *μ*m large area was irradiated with the electron beam (*E*_kin_ = 800 eV, 1 nA) of a SEM (CS44 CamScan) with no gas inlet for 96 hours. The chamber pressure was 5•10^−6^ mbar. After the irradiation process the mask was removed in an ultrasonic bath of acetone. The following annealing and etching processes were under the same conditions as before and the subsequently recorded fluorescence images and spectra were taken with the aforementioned confocal microscope.
